# Association of elevated apoA-I glycation and reduced HDL-associated paraoxonase1, 3 activity, and their interaction with angiographic severity of coronary artery disease in patients with type 2 diabetes mellitus

**DOI:** 10.1186/s12933-015-0221-4

**Published:** 2015-05-13

**Authors:** Ying Shen, Feng Hua Ding, Jia Teng Sun, Li Jin Pu, Rui Yan Zhang, Qi Zhang, Qiu Jing Chen, Wei Feng Shen, Lin Lu

**Affiliations:** Department of Cardiology, Rui Jin Hospital, Shanghai Jiaotong University School of Medicine, 197 Rui Jin Road II, Shanghai, 200025 People’s Republic of China; Institute of Cardiovascular Disease, Shanghai Jiaotong University School of Medicine, Shanghai, People’s Republic of China

**Keywords:** Diabetes mellitus, ApoA-I glycation, Paraoxonase, Coronary artery disease

## Abstract

**Objective:**

To investigate whether apolipoprotein A (apoA)-I glycation and paraoxonase (PON) activities are associated with the severity of coronary artery disease (CAD) in patients with type 2 diabetes mellitus (T2DM).

**Methods:**

Relative intensity of apoA-I glycation and activities of high-density lipoprotein (HDL)-associated PON1 and PON3 were determined in 205 consecutive T2DM patients with stable angina with (n = 144) or without (n = 61) significant CAD (luminal diameter stenosis ≥ 70 %). The severity of CAD was expressed by number of diseased coronary arteries, extent index, and cumulative coronary stenosis score (CCSS).

**Results:**

The relative intensity of apoA-I glycation was higher but the activities of HDL-associated PON1 and PON3 were lower in diabetic patients with significant CAD than in those without. The relative intensity of apoA-I glycation increased but the activities of HDL-associated PON1 and PON3 decreased stepwise from 1 - to 3 - vessel disease patients (*P* for trend < 0.001). After adjusting for possible confounding variables, the relative intensity of apoA-I glycation correlated positively, while the activities of HDL-associated PON1 and PON3 negatively, with extent index and CCSS, respectively. At high level of apoA-I glycation (8.70 ~ 12.50 %), low tertile of HDL-associated PON1 (7.03 ~ 38.97U/mL) and PON3 activities (7.11 ~ 22.30U/mL) was associated with a 1.97− and 2.49− fold increase of extent index and 1.73− and 2.68− fold increase of CCSS compared with high tertile of HDL-associated PON1 (57.85 ~ 154.82U/mL) and PON3 activities (39.63 ~ 124.10U/mL), respectively (all *P* < 0.01).

**Conclusions:**

Elevated apoA-I glycation and decreased activities of HDL-associated PON1 and PON3, and their interaction are associated with the presence and severity of CAD in patients with T2DM.

**Electronic supplementary material:**

The online version of this article (doi:10.1186/s12933-015-0221-4) contains supplementary material, which is available to authorized users.

## Introduction

It is well recognized that high-density lipoprotein (HDL) exerts a protective effect on cardiovascular system, and serum HDL-cholesterol (HDL-C) levels are negatively associated with the risk of coronary artery disease (CAD), metabolic syndrome and insulinresistance [1,2]. The anti-atherogenic properties of HDL are mainly related to reverse cholesterol transport, stabilization of atherosclerotic plaque, and anti-inflammatory and anti-oxidant effects [1, 3]. Apolipoprotein A-I (apoA-I) accounts for approximately 70% of the total protein mass of HDL, and the remaining protein components mainly include apoA-II, apoC, apoA-IV, and paraoxonase (PON) [4]. The PON family comprises three members: PON1, PON2 and PON3, among which PON1 and PON3 are almost exclusively associated with HDL [5]. Both PON 1 and PON 3 prevent low-density lipoprotein cholesterol (LDL-C) from peroxidation, conferring antagonistic effects against atherosclerosis [6, 7]. Normal apolipoproteins in HDL, mainly apoA-I, contribute to enzyme activity, stability, and function of PON [8, 9]. Animal experiments of apoA-I deficient mice have shown that an accelerated atherosclerotic process is mechanistically attributed to impaired reverse cholesterol transport, reduced PON activity, and augmented inflammation [10], whereas peritoneal injection of apoA-I mimetic peptide effectively prevents early atherogenesis, accompanied with increment of PON activity [11].

In type 2 diabetes mellitus (T2DM), vascular complications are mainly due to prolonged exposure to hyperglycemia clustering with other diseases such as hypertension and dyslipidemia and other risk factors including retinol-binding protein-4 and hypoxia-induced factor 1α [12–14]. Besides, the formation of advanced glycation end products (AGEs) has been implicated in coronary atherogenesis [15–17]. Glycation of apoA-I significantly impairs anti-inflammatory and anti-atherogenic properties of HDL [18], and is associated with coronary plaque progression [19]. In contrast, inhibition of such glycation rescues HDL function [20], and infusion of reconstituted HDL increases cholesterol efflux and reduces atherosclerotic plaque volume [21, 22]. To the best of our knowledge, PON activity, especially the activity of HDL-associated PON, and the interactive effect of apoA-I glycation and HDL-associated PON activity on coronary atherosclerosis in T2DM remain unknown. In this study, we measured apoA-I glycation level and serum and HDL-associated activities of PON1 and PON3, to test the hypothesis that elevated apoA-I glycation and reduced PON1 and PON3 activities and their interaction were related to the presence and severity of CAD in patients with T2DM.

## Methods

The research protocol was approved by the Institutional Review Board of Rui Jin Hospital, Shanghai Jiaotong University School of Medicine, and was registered (NCT02089360). Informed consent was obtained in written form from all patients, and clinical investigation was conducted according to the principle of the Declaration of Helsinki.

### Study population

A total of 317 consecutive patients with T2DM and chest pain on exertion referred for diagnostic coronary angiography between July 2012 and November 2013 were enrolled. Baseline demographics, risk factors for CAD, and medications were recorded. The diagnosis of T2DM was made according to the criteria of the American Diabetes Association [23], including a fasting blood glucose (FBG) level of ≥7.0 mmol/L or 2-h postprandial plasma glucose (2 h-PG) readings ≥11.1 mmol/L by multiple determinations or currently receiving insulin or oral hypoglycemic agents. Hypertension and dyslipidemia were diagnosed according to seventh report of the Joint National Committee on prevention, detection, evaluation, and treatment of high blood pressure (JNC 7) and guideline of the National Cholesterol Education Program (ATP III), respectively [24, 25].

For the purpose of research, patients with acute coronary syndrome (n = 41) or a history of coronary revascularization (coronary artery bypass grafting: n = 6; percutaneous coronary intervention: n = 21) were excluded. We also excluded patients with renal failure requiring hemodialysis (n = 2) and those who had chronic heart failure, pulmonary heart disease, malignant tumor or immune system disorders (n = 37). Patients with type 1 diabetes were excluded by measurement of C-peptide (n = 5). The remaining 205 eligible patients were included in the final analysis of this study.

### Coronary angiography and analysis

Coronary angiography was performed through radial or femoral approach. Significant CAD was diagnosed if luminal diameter narrowing was estimated visually as ≥ 70 % in a major epicardial coronary artery [26]. Left main coronary artery stenosis ≥ 50 % was considered as 2 - vessel disease. Quantitative coronary angiography (QCA) was performed using the Cardiovascular Measurement System version 3.0 software (Terra, GE, USA) by two interventional cardiologists who were blinded to the study protocol. The extent index and cumulative coronary stenosis score (CCSS) were used as indices of the anatomic extension and severity of CAD. The extent index was calculated as the longitudinal percentage of coronary segments involved with a stenosis (∑ [stenosis lengths]/∑ [segment lengths]) [15]. The CCSS was calculated as previously reported by adding all percent diameter stenosis in stenosis index units (50 % = 0.50) [27].

### Biochemical measurements

Blood samples were obtained after an overnight fasting in all participants. Serum levels of glucose, blood urea nitrogen, creatinine, uric acid, total cholesterol, HDL cholesterol, LDL-C, lipoprotein (a), apoproteinA, apoprotein B, and triglycerides were measured with standard laboratory techniques on a Hitachi 912 Analyzer (Roche Diagnostics, Germany). Blood concentration of glycosylated hemoglobin (HbA1c) was assayed using ion-exchange high performance liquid chromatography with a Bio-Rad Variant Hemoglobin Testing System (Bio-Rad Laboratories, Hercules, CA, USA). Serum levels of PON1 and PON3 were determined using commercially available ELISA kit (MyBioSource, Canada). High-sensitivity C-reactive protein (hsCRP) level was assessed by ELISA (Biocheck Laboratories, Toledo, OH, USA).

### Isolation of HDL fraction and SDS-polyacrylaminde gel electrophoresis

Human serum HDL (1.063 < d <1.21 g/L) was isolated from fresh plasma by ultracentrifugation using potassium bromide method as described previously [28]. Then, HDL fraction was dialyzed and separated by SDS-polyacrylamide gel electrophoresis. ApoA-I protein could be visualized after silver nitrate staining.

### Western blot analysis of apoA-I glycation

HDL fraction separated by SDS-polyacrylamide gel electrophoresis was transferred to polyvinylidene fluoride membrane. After blocking with 5 % milk, the membrane was incubated overnight at 4 °C with anti-apoA-I (Santa cruz biotechnology) oranti-Nε-(carboxyethyl)-lysine (CEL) antibody (Cosmo Bio Co., Tokyo, Japan). Then, ECL reagent (GE Healthcare, UK) was used for detection. Films were examined using an HP Scanjet Pro flatbed scanner, and images were analyzed and quantified with Adobe Photoshop CS2 software. Absolute intensity was assessed via multiplying the mean density value by pixel for each band, and relative intensity of apoA-I glycation was calculated as absolute intensity of apoA-I glycation divided by that of apoA-I protein.

### Measurement of serum and HDL-associated PON1, 3 activities

PON1 arylesterase activity was analyzed in serum and in HDL with phenyl acetate as a synthetic substrate [29]. The assay mixture contained 100 μl of 10 mmol/L substrate solution, 5 μL serum and 1 mmol/L CaCl2 in 50 mmol/L Tris buffer. Production of phenol was determined spectrophotometrically after 2 min at 270 nm. PON1 arylesterase activity was monitored in triplicate and the results are presented as μmoL/min per mL (U/mL). Serum and HDL-associated PON3 statinase activity (the hydrolysis of lovastatin lactones) was determined by HPLC. In a final volume of 1 mL, 100 μL of enzyme and 10 μL of substrate solution in methanol (0.5 mg/mL) were incubated at 25 °C in 25 mM Tris/HCl, 1 mM CaCl_2_. Aliquots (100 μL) were removed at specific times and added to cold acetonitrile (100 μL), mixed and the supernatants were subjected to HPLC analysis at wavelength 238 nm. Samples were eluted at a flow rate of 1.0 mL/min with a mobile phase consisting of the following: A = acetic acid/acetonitrile/water (2:249:249, v/v/v) and B = acetonitrile, in A/B ratios of 35/65. PON3 statinase activity was repeated in triplicate and the results are presented as pmol lovastatin hydrolyzed per min per mL (U/mL).

### Statistical analyses

Data are expressed as mean ± standard deviation for continuous variables, and frequencies and percentages for categorical ones. For continuous variables, the existence of a normal distribution was ascertained by the Kolmogorov–Smirnov test. For multiple comparisons between groups, one way analysis of variance (ANOVA) was used followed by the Bonferoni’s method. Proportions were compared byΧ^2^test or Fisher’s exact test when appropriate. Pearson’s and Spearman’s correlation tests were used to assess the relation between variables. Receiver-operating characteristic (ROC) analyses were used to determine the power of the relative intensity apoA-I glycation and activities of PON for detecting significant CAD, and the areas under the curve were compared using the DeLong method. Multivariable linear regression analyses were performed to assess the independent determinants of extent index and CCSS after adjusting for possible confounding factors including gender, age, body mass index (BMI), traditional risk factor for CAD, HbA1c, total/HDL cholesterol ratio, estimated glomerular filtration rate (eGFR), hsCRP, and use of statins. SPSS 20.0 software (SPSS Inc, Chicago, Illinois, USA) was used for all statistical testing. A 2-tailed < 0.05 was considered statistically significant.

## Results

### Baseline characteristics

Clinical features and biochemical measurements are listed in Table 1. Male, hypertension and smoking were higher in proportion, and serum levels of creatinine, fasting blood glucose, HbA1c, and hsCRP were more elevated, but HDL-C level was lower in T2DM patients with significant CAD than in those without (for all comparisons, *P* < 0.05). Medications were comparable between the two groups except that more patients with significant CAD received insulin therapy.

**Table 1:** Baseline characteristics and biochemical assessments in type 2 diabetic patients

Variables	CAD (+)	CAD (−)	*P* value
	(n = 144)	(n = 61)	
Male, n (%)	108 (75.0)	37 (60.7)	0.039
Age, years	64.8 ± 10.3	65.3 ± 9.1	0.728
Body mass index, Kg/m^2^	25.5 ± 3.4	24.9 ± 3.1	0.262
Smoking, n (%)	50 (34.7)	11 (18.0)	0.017
Hypertension (%)	110 (76.4)	37 (60.7)	0.022
Systolic blood pressure, mmHg	140 ± 19	139 ± 20	0.572
Diastolic blood pressure,mmHg	81 ± 12	81 ± 11	0.603
Dyslipidemia history, n (%)	57 (39.6)	23 (37.7)	0.801
Triglycerides, mmol/L	1.82 ± 1.09	1.75 ± 0.96	0.671
Total cholesterol, mmol/L	4.3 ± 1.2	4.4 ± 1.0	0.753
HDL cholesterol, mmol/L	1.01 ± 0.24	1.13 ± 0.22	0.001
LDL cholesterol, mmol/L	2.63 ± 0.93	2.56 ± 0.76	0.616
Lipoprotein (a), g/L	0.27 ± 0.25	0.28 ± 0.26	0.666
Apoprotein A, g/L	1.20 ± 0.19	1.23 ± 0.20	0.310
Apoprotein B, g/L	0.91 ± 0.26	0.88 ± 0.20	0.460
Serum creatinine, μmol/L	81 ± 17	75 ± 14	0.011
eGFR, mL/min/1.73 m^2^	92.3 ± 24.4	96.8 ± 23.6	0.228
Uric acid, μmol/L	342 ± 89	325 ± 83	0.199
Fasting blood glucose, mmol/L	6.79 ± 2.00	6.02 ± 1.33	0.001
HbA1c, %	7.81 ± 1.07	6.89 ± 0.75	<0.001
hsCRP, mg/L	6.20 ± 3.24	5.21 ± 2.42	0.040
Extent index	0.48 ± 0.13	0.27 ± 0.08	<0.001
Cumulative coronary stenosis score	2.17 ± 0.71	0.92 ± 0.42	<0.001
Relative intensity of apoA-I glycation, %	8.27 ± 2.09	5.69 ± 1.42	<0.001
Activities of paraoxonase, U/mL			
Serum PON1 activity	78.46 ± 16.94	86.01 ± 17.36	0.004
HDL-associated PON1 activity	41.43 ± 14.64	92.19 ± 33.42	<0.001
Serum PON3 activity	32.15 ± 5.33	39.2 ± 6.62	<0.001
HDL-associated PON3 activity	26.06 ± 12.66	64.58 ± 22.44	<0.001
Medical treatments, n (%)			
Insulins	51 (35.4)	11 (18.0)	0.013
Metformin	70 (48.6)	31 (50.8)	0.772
Sulphonylurea	41 (28.5)	25 (41.0)	0.080
α-Glucosidase	40 (27.8)	18 (29.5)	0.801
Statin	107 (74.3)	43 (70.5)	0.573
ACE inhibitor/ARB	88 (61.1)	37 (60.7)	0.951
β-blocker	82 (56.9)	34 (55.7)	0.873
Calcium channel blocker	73 (50.7)	39 (63.9)	0.082
Antiplatelet	107 (74.3)	36 (59.0)	0.029

Data are mean ± SD and number (%)

ACE, angiotensin-converting enzyme; apoA-I, apolipoprotein A-I; ARB, angiotensin receptor blocker; CAD, coronary artery disease; eGFR, estimated glomerular filtration rate; HbA1c, glycated hemoglobinA1c; HDL, high-density lipoprotein; hsCRP, high-sentivity C-reactive protein; LDL, low densitylipoprotein, PON, paraoxonase

### Relation of apoA-I glycation and PON activity with CAD in patients with T2DM

The relative intensity of apoA-I glycation was higher but the activities of HDL-associated PON1 and PON3 were lower in diabetic patients with significant CAD than in those without. Consistently, the relative intensity of apoA-I glycation increased but the activities of HDL-associated PON1 and PON3 decreased stepwise from 1- to 3-vessel disease patients (*P* for trend < 0.001). After adjusting for age, gender, BMI, history of hypertension and dyslipidemia, smoking, HbA1c, estimated glomerular filtration rate, total/HDL cholesterol ratio, hsCRP and statin use, relative intensity of apoA-I glycation correlated positively, while HDL-associated PON1 and PON3 activities negatively, with extent index and CCSS, respectively (all *P* < 0.001) (Table 2). In addition, the relative intensity of apoA-I glycation was inversely related to the activities of HDL-associated PON1 and PON3 (r = −0.252 and −0.478, all *P* < 0.001). The relation pattern was similar for serum activities of PON1 and PON3. ROC curve analysis confirmed the value of relative intensity of apoA-I glycation and serum and HDL-associated PON1 and PON3 activities in evaluating the presence and severity of CAD (Table 3). However, the areas under the curve of HDL-associated PON1 and PON3 activities were significantly larger than those of serum PON1 and PON3 activities (Additional file 1: Figure S1).

**Table 2:** Correlation of apoA-I glycation and serum and HDL-associated PON1, 3 activities with the severity of CAD in patients with type 2 diabetes

Variables	Relative intensity of apoA-I glycation (%)	Serum PON1 activity (U/mL)	HDL-associated PON1 activity (U/mL)	Serum PON3 activity (U/mL)	HDL-associated PON3 activity (U/mL)
Diseased vessels					
0-vessel disease (n = 61)	5.69 ± 1.43	86.01 ± 17.36	92.19 ± 33.43	39.21 ± 6.62	64.58 ± 22.44
1-vessel disease (n = 41)	5.98 ± 1.08	85.96 ± 17.17	48.12 ± 11.34	35.81 ± 5.20	38.76 ± 12.78
2-vessel disease (n = 53)	8.17 ± 1.26	79.06 ± 16.10	42.19 ± 14.37	32.51 ± 5.65	22.07 ± 7.58
3-vessel disease (n = 50)	10.24 ± 1.32	71.67 ± 15.09	35.15 ± 14.94	31.25 ± 5.80	19.88 ± 9.06
Unadjusted Spearman r	0.800	−0.305	−0.659	−0.500	−0.782
Unadjusted P for trend	<0.001	<0.001	<0.001	<0.001	<0.001
*Adjusted Spearman r	0.730	−0.345	−0.562	−0.414	−0.691
*Adjusted P for trend	<0.001	<0.001	<0.001	<0.001	<0.001
Extent index					
Unadjusted r	0.627	−0.278	−0.588	−0.358	−0.600
Unadjusted P	<0.001	<0.001	<0.001	<0.001	<0.001
*Adjusted r	0.546	−0.291	−0.508	−0.296	−0.537
*Adjusted P	<0.001	<0.001	<0.001	<0.001	<0.001
Cumulative coronary stenosis score					
Unadjusted r	0.709	−0.243	−0.572	−0.358	−0.594
Unadjusted P	<0.001	<0.001	<0.001	<0.001	<0.001
*Adjusted r	0.626	−0.267	−0.453	−0.288	−0.488
*Adjusted P	<0.001	<0.001	<0.001	<0.001	<0.001

Values are means ± SD or Peason corrlation coefficients unless otherwise indicated

CAD, coronary artery disease; HDL, high-density lipoprotein; PON, paraoxonase

*adjusted for gender, age, body mass index, history of hypertension and dyslipidemia, smoking, glycated hemoglobin A1c, estimated glomerular filtration rate, total/HDL cholesterol ratio, high-sentivity C-reactive protein and use of statins

**Table 3:** Value of apoA-I glycation and serum and HDL-associated PON1, 3 activities in evaluating severity of CAD in type 2 diabetes

Activities of paraoxonase	Significant CAD		Multi-vessel disease		High tertile of extent index		High tertile of CCSS	
	AUC (95 % CI)	*P* value	AUC (95 % CI)	*P* value	AUC (95 % CI)	*P* value	AUC (95 % CI)	*P* value
Relative intensity of apoA-I glycation	0.849 (0.792 ~ 0.906)	<0.001	0.952 (0.922 ~ 0.981)	<0.001	0.855 (0.806 ~ 0.904)	<0.001	0.893 (0.843 ~ 0.943)	<0.001
Serum PON1 activity	0.617 (0.533 ~ 0.702)	0.008	0.664 (0.590 ~ 0.737)	<0.001	0.598 (0.512 ~ 0.684)	0.022	0.599 (0.518 ~ 0.680)	0.021
HDL-associated PON1 activity	0.908 (0.857 ~ 0.960)*	<0.001	0.828 (0.774 ~ 0.883)*	<0.001	0.824 (0.760 ~ 0.888)*	<0.001	0.823 (0.759 ~ 0.887)*	<0.001
Serum PON3 activity	0.765 (0.694 ~ 0.837)	<0.001	0.777 (0.712 ~ 0.841)	<0.001	0.693 (0.618 ~ 0.769)	<0.001	0.743 (0.672 ~ 0.814)	<0.001
HDL-associated PON3 activity	0.941 (0.909 ~ 0.973)#	<0.001	0.939 (0.908 ~ 0.970)#	<0.001	0.866 (0.812 ~ 0.921)#	<0.001	0.881 (0.835 ~ 0.927)#	<0.001

AUC, area under the curve; CAD, coronary artery disease; CCSS, cumulative coronary stenosis score; CI, conficence interval; HDL, high-density lipoprotein; PON, paraoxonase

*P < 0.001 vs. corresponding AUCs of serum PON1 activity; #*P* < 0.001 vs. corresponding AUCs of serum PON3 activity

### Multivariable analysis

After adjustment for confounding variables, the association between the relative intensity of apoA-I glycation and the activities of HDL-associated PON1 (model 1) or PON3 (model 2) with extent index and CCSS (adjusted R^2^) was increased if an interaction between the relative intensity of apoA-I glycation and the activities of HDL-associated PON1 or PON3 was considered (Table 4). Meanwhile, the activities of HDL-associated PON1 or PON3 (*P* ≥ 0.179 and *P* ≥ 0.124) were replaced by the interaction between the relative intensity of apoA-I glycation and HDL-associated PON1 (*P* < 0.001 and *P* = 0.004) or PON3 activities (*P* = 0.019 and *P* < 0.001) for extent index and CCSS, respectively.

**Table 4:** Extent index and cumulative coronary stenosis score in relation to apoA-I glycation and HDL-associated PON1, 3 activities

			Extent index			Cumulative coronary stenosis score		
			Adjusted R^2^	β ± SE	P value	Adjusted R^2^	β ± SE	P value
Model 1	A	Relative intensity of apoA-I glycation	0.589	0.407 ± 0.004	< 0.001	0.636	0.483 ± 0.022	< 0.001
		HDL-associated PON1 activity		−0.439 ± 0.000	< 0.001		−0.302 ± 0.002	< 0.001
	B	Relative intensity of apoA-I glycation	0.627	0.760 ± 0.007	< 0.001	0.645	0.666 ± 0.039	< 0.001
		HDL-associated PON1 activity		0.207 ± 0.001	0.179		0.033 ± 0.004	0.827
		*Interaction		−0.603 ± 0.000	< 0.001		−0.312 ± 0.001	0.019
Model 1	A	Relative intensity of apoA-I glycation	0.541	0.395 ± 0.005	< 0.001	0.634	0.448 ± 0.023	< 0.001
		HDL-associated PON3 activity		−0.383 ± 0.000	< 0.001		−0.320 ± 0.002	< 0.001
	B	Relative intensity of apoA-I glycation	0.558	0.623 ± 0.008	< 0.001	0.658	0.707 ± 0.037	< 0.001
		HDL-associated PON3 activity		0.116 ± 0.001	0.523		0.247 ± 0.06	0.124
		*Interaction		−0.441 ± 0.000	0.004		−0.500 ± 0.001	< 0.001

Values are regression coefficients (β) ± standard error (SE)

The relative intensity of apoA-I glycation in addition with HDL-associated PON1, 3 activity were included (Model 1A and Model 2A). The interactions between the relative intenstity of apoA-I glycation and HDL-associated PON1, 3 activities were further included (Model 1B andModel 2B). All models were adjusted for gender, age, body mass index, history of hypertension and dyslipidemia, smoking, glycatedhemoglobin A1c, estimated glomerular filtration rate, total/HDL cholesterol ratio, high-sentivity C-reactive protein and use of statins. apoA-I, apolipoprotein A-I; HDL, high-density lipoprotein; PON, paraoxonase

*Interaction denote the interaction between relative intenstity of apoA-I glycation and HDL-associated paraoxonase activities. Extent indexCumulative coronary stenosis scoreModel 1

**Figure 1:**
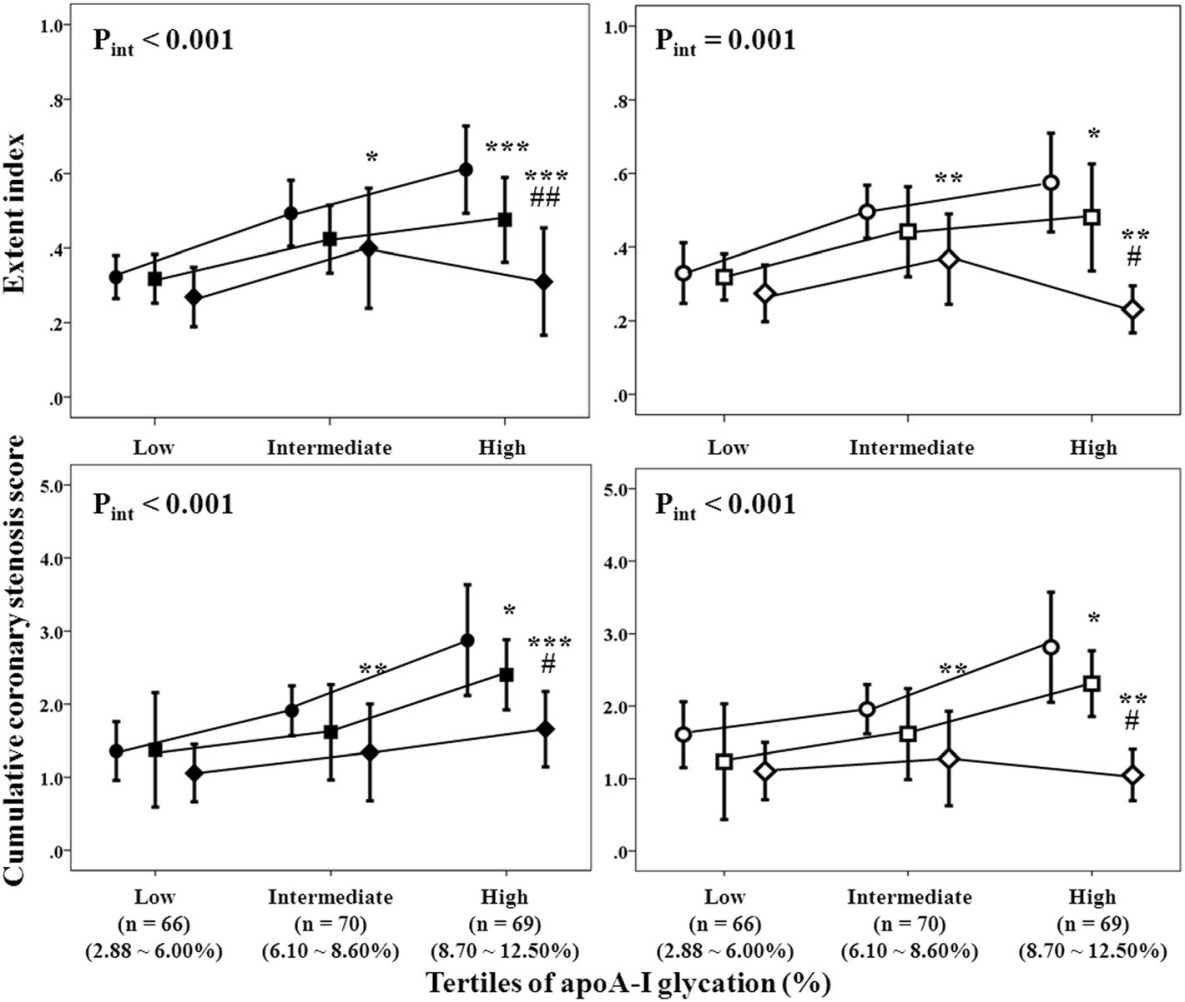
Extent index and cumulative coronary stenosis score in relation to apoA-I glycation and HDL-associated activities of PON1 and PON3. Tertiles of HDL-associated activities of PON1 (●low [7.03 ~ 38.97 U/mL], ■middle [40.37 ~ 57.17 U/mL], ♦high [57.85 ~ 154.82 U/ml]) and PON3 (○low [7.11 ~ 22.30 U/mL], □middle [23.12 ~ 39.57 U/mL], ◊high [39.63 ~ 124.10 U/mL]).*P < 0.05, **P < 0.01, ***P < 0.001 vs. low tertile of activities of PON1 or PON3; #*P* < 0.05, ##*P* < 0.01 vs. middle tertile of activities of PON1 or PON3

Patients were then reclassified according to tertile distribution of apoA-I glycation and the activities of HDL-associated PON1 and PON3. At high level of apoA-I glycation (8.70 ~ 12.50 %), patients with low tertile of HDL-associated PON1 (7.03 ~ 38.97 U/mL) and PON3 activities (7.11 ~ 22.30 U/mL) had 1.97− and 2.49− fold increase of extent index and 1.73− and 2.68− fold increase of CCSS compared with those with high tertile of HDL-associated PON1 and PON3 activities, respectively (all *P* < 0.01). Similar pattern was observed in patients with intermediate (6.10 ~ 8.60 %) but not low tertile of apoA-I glycation (2.88 ~ 6.00 %) (Fig. 1 and Additional file 2: Table S1). However, there was no interaction between relative intensity of apoA-I glycation and serum activities of PON1 and PON3 on the severity of CAD (Additional file 2: Tables S1 and Additional file 3: Tables S2).

## Discussion

The present study has demonstrated that glycation of apoA-I was associated with decreased activities of serum and HDL-associated PON1 and PON3. Elevated apoA-I glycation and reduced HDL-associated PON1 and PON3 activities, and the interaction of these two elements were related to the presence and severity of CAD in patients with T2DM.

HDL is an organized complex of proteins (apo and enzyme) and lipids (cholesterol, cholesteryl ester, triglyceride, and phospholipid), and possesses several functions with potential to protect against coronary atherosclerosis, by promoting efflux of cholesterol from macrophages in the arterial wall, inhibiting oxidative modification of low density lipoprotein, decreasing vascular inflammation, enhancing endothelial repair, and improving diabetic control [1, 2]. The structural and functional integrity of apoA-I is crucial for the activation and stability of lecithin:cholesterol acyl transferase (LCAT) and PON [10, 11]. Mutation, glycation and oxidative modification of apoA-I markedly impair the ability of apoA-1 to act as substrates for LCAT and promote pathogenesis [30–32]. Both PON1 and PON3 are almost exclusively associated with HDL, and reduced function of PON cripples their protection of lipoproteins against oxidative modifications [33]. Previous studies have consistently reported that PON1 is a marker of cardiovascular risk in youth with type 1 diabetes and that Q192R polymorphism of PON 1 gene is associated with insulin resistance [34, 35].

The main finding of this study is that relative intensity of apoA-I glycation was elevated but serum and HDL-associated PON1 and PON3 activities were reduced in T2DM patients with significant CAD. This is consistent with previous reports that advanced glycation of apoA-I impairs its anti-atherogenic properties [18], and is associated with decreased LCAT activity and coronary atherosclerotic plaque progression in patients with T2DM [19]. Furthermore, we found that relative intensity of apoA-I glycation correlated positively, while HDL-associated PON1 and PON3 activities inversely, with the severity of coronary disease assessed by number of diseased coronary arteries, extent index, and CCSS even after adjusting for possible confounding factors. Interestingly, when an interaction between relative intensity of apoA-I glycation and HDL-associated PON1 and PON3 activities was introduced into the multivariable regression models, adjusted R^2^ was significantly increased for both extent index and CCSS. At middle and particularly high tertile of apoA-I glycation, patients with low levels of HDL-associated activities of PON1 and PON3 had approximately 2 to 2.5 fold increased risk for severe coronary atherosclerosis. However, such a relation between PON activities and severity of CAD was not observed at the low tertile of apoA-1 glycation. These results support a notion that severely impaired HDL function caused by moderate or high degree of major apolipoprotein glycation in HDL (e.g., apoA-I), together with reduced PON activities possibly due to apolipoprotein glycation, are important in accelerating the process of coronary atherosclerosis in T2DM.

### Study limitations

We recognize limitations in our findings. The most relevant one is that the study presented here is cross-sectional, thereby allowing us to detect associations, but not to infer causality or to formulate predictions. Larger-scale, long-term prospective studies are needed to confirm our results and to assess the prognostic significance of possible medications that inhibit apo A-I glycation and increase PON activities. Finally, the classification of significant CAD based on visual estimation of angiographic stenosis of coronary artery lesions at ≥70 % is admittedly arbitrary. However, within the range of angiographically significant CAD, including lesions of ≥70 % stenosis, this criterion of severity correlates well with physiological standards and is widely accepted clinical practice [26].

## Conclusions

This study indicates that elevated apoA-I glycation and reduced serum and HDL-associated PON activities, and their interaction are associated with the presence and severity of stable CAD in patients with T2DM.

## Additional files

Additional file 1: Figure S1. Receiver operating characteristic curves of serum (dash lines) and high-density lipoprotein associated (solid lines) activity of paraoxonase1 (grey lines) and 3 (black lines) for evaluating presence and severity of coronary artery disease (CAD), including significant CAD (A) and multi-vessel disease (B) and high tertile of extent index (C) and cumulative coronary stenosis score (D), in type 2 diabetes mellitus. HDL, high-density lipoprotein; PON, paraoxonase. Additional file 2: Table S1. Extent index and cumulative coronary stenosis score in relation to interactions between apoA-I glycation and serum and HDL-associatedactivities of PON1 and PON3. Additional file 3: Table S2. Extent index and cumulative coronary stenosis score in relation to apoA-I glycation and serum activities ofPON1 and PON3.
