# Supplementary figures and images for: Association of elevated apoA-I glycation and reduced HDL-associated paraoxonase1, 3 activity, and their interaction with angiographic severity of coronary artery disease in patients with type 2 diabetes mellitus
Source: Cardiovasc Diabetol. 2015 May 13;14:52. doi: 10.1186/s12933-015-0221-4 (PMC4432963; doi:10.1186/s12933-015-0221-4)

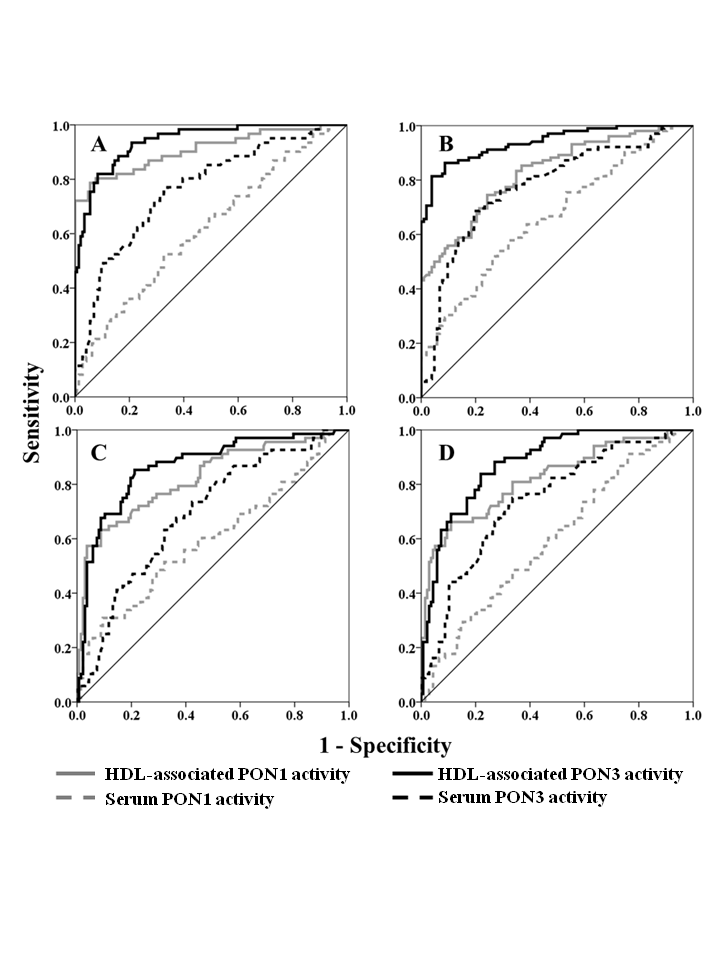

Supplement: Additional file 1: Figure S1. — Receiver operating characteristic curves of serum (dash lines) and high-density lipoprotein associated (solid lines) activity of paraoxonase1 (grey lines) and 3 (black lines) for evaluating presence and severity of coronary artery disease (CAD), including significant CAD (A) and multi-vessel disease (B) and high tertile of extent index (C) and cumulative coronary stenosis score (D), in type 2 diabetes mellitus. HDL, high-density lipoprotein; PON, paraoxonase. [file 12933_2015_221_MOESM1_ESM.zip › 12933_2015_221_MOESM1_ESM.bmp]
