# Supplementary material for: Association of elevated apoA-I glycation and reduced HDL-associated paraoxonase1, 3 activity, and their interaction with angiographic severity of coronary artery disease in patients with type 2 diabetes mellitus
Source: Cardiovasc Diabetol. 2015 May 13;14:52. doi: 10.1186/s12933-015-0221-4 (PMC4432963; doi:10.1186/s12933-015-0221-4)
Supplement: Supplementary file 4 [file 12933_2015_221_MOESM3_ESM.docx]

Supplementary Table 2. Extent index and

and PON3.
